# Iodine-131 induced hepatotoxicity in previously healthy patients with Grave’s disease

**DOI:** 10.1186/1756-6614-6-4

**Published:** 2013-03-16

**Authors:** Navina Priya Jhummon, Bhavna Tohooloo, Shen Qu

**Affiliations:** 1 Department of Endocrinology and Metabolism, Tenth People’s Hospital of Tongji University, Tongji University, School of Medicine, Shanghai, 200072, China

**Keywords:** Hepatotoxicity, ^131^I, Radioactive iodine, RAI, Grave’s disease, GD

## Abstract

**Objective:**

To describe the association of the rare and serious complication of liver toxicity in previously healthy Grave’s disease (GD) patients after the treatment with radioactive iodine ^131^I (RAI).

**Case presentation:**

We report the clinical, laboratory and pathologic findings of 2 cases of severe liver toxicity associated with the treatment with RAI in previously healthy patients with GD. Clinical examination and laboratory investigations excluded viral hepatitis, autoimmune hepatitis, granulomatous disease, primary biliary disease, extrahepatic biliary obstruction, and heart failure.

Case 1: A previously healthy 52-years old man reportedly having a typical GD but following RAI treatment, concomitantly developed severe liver toxicity that required 1 week of treatment in hospital.

Case 2: A previously healthy 34-years old woman is reported as having a typical GD but developed jaundice following RAI treatment that required several weeks of in hospital treatment in the hepato-biliary department.

In both cases, the liver dysfunction resolved after intensive treatment with hepato-protective agents. In this report the therapeutic considerations as well as the pathogenetic possibilities are reviewed.

**Conclusion:**

To the best of our knowledge, this is the first description of the association observed, which is rare but may be severe and should be considered in any case of thyrotoxicosis where a liver dysfunction develops after the treatment with radioactive iodine ^131^I.

## Introduction

Graves’ disease is the most common cause of hyperthyroidism in iodine-sufficient areas [1]. Several therapeutic options are available for the treatment of hyperthyroidism due to GD, including antithyroid drug medication (ATD), near-total resection (NTR) and radioiodine therapy (RIT). Each therapeutic option has its advantages and disadvantages [2].

Oral administration of ^131^I has been used to treat benign conditions of the thyroid gland since the 1940s [3]. In our hospital, the treatment with RAI was initiated in 1983. The aim of treatment with ^131^I is to achieve a non-hyperthyroid status, which can be euthyroid or hypothyroid, recompensated by levothyroxine medication [3]. RAI treatment is widely used because it is easy to be administered, widely available, and effective in most patients [4]. To date, our nuclear medicine department welcomes an average of 350 patients yearly for RIT.

The usual treatment in our hospital is with a fixed activity regimen that ranges between 5-7 mCi with a mean effective half life (EHL) of 7.2 days.

Liver enzyme elevations in thyroid disease might be paradoxically related to RIT. However, here we present two cases of liver toxicity developing in previously healthy Grave’s disease patients when treated with RAI, and subsequent improvement via hepato-protective treatment regimens. Our aim is to draw the attention of physicians to hyperthyroidism and its treatment with RAI in the differential diagnosis of liver dysfunction.

## Case presentation

### Case 1

A 52-year-old male patient was diagnosed with GD 20-years ago with history of heat intolerance, sweating, weight loss, tremors, increased appetite, accompanied by change in temperament, and exophthalmos. The patient was put on a treatment with anti-thyroid drug (ATD) propylthiouracil, methimazole and prednisolone which brought an improvement of the symptoms, exophthalmos, and thyroid function. However, some 5-years ago, he noticed an enlargement of the thyroid and 6-months later, he complained of persistent profuse sweating, heat intolerance, palpitation, associated with neck pain, mild breathing difficulty, there were no significant weight loss and no significant increase in bowel movements. He was put on methimazole (10 mg bid), which proved ineffective. He denied having any history of smoking, alcohol or drug abuse. There was no significant past medical or surgical history.

He was admitted in our department in July 2012 and upon physical examination, his blood pressure was 120/82 mmHg, and heart rate of 98/min regular. He had moist skin, tremors, bilateral moderate exophthalmos of mean and no pedal oedema. His thyroid showed a bilateral 3rd degree enlargement, soft in consistency, non tender, no palpable nodules and no bruits upon auscultation. Thyroid ultrasonography showed bilateral enlargement of the thyroid gland and radioactive iodine uptake test showed a significantly elevated thyroid ^123^I uptake function at 2 and 24 hours of 95.7% and 71.4% respectively. Orbital CT showed bilateral exophthalmos; the prominence was of 19.0 mm and 19.3 mm Hertel readings on the right and left sides respectively, abdominal ultrasound showed no abnormalities. Blood chemical tests documented normal renal function and hepatic values but revealed a significant increase in serum thyroid hormone levels (Table 1). The possibility of primary liver disease could be ruled out; there was no evidence of immunological conditions (autoimmune hepatitis, primary sclerosing cholangitis or primary biliary cirrhosis); neither was metabolic liver disease due to alpha-1 antitrypsin deficiency, hemochromatosis or Wilson´s disease identified. Hepatitis serology was negative and serology for several other virus infections that might lead to liver dysfunction did not indicate presence of active virus infection. The patient´s alcohol consumption was sparse limited to less than one drink a month. Grave’s hyperthyroidism was diagnosed and the treatment dosage for the patient was determined by the intake rate and the size of thyroid gland. If the dosages is over 10 mCi ^131^I, the patients will be treated in two divided doses of 5–7 mCi each to minimise side-effects.

**Table 1:** Chronological thyroid function test and liver function test for case 1

**Case 1**									
**Event**	**fT3**	**fT4**	**TSH**	**Tb**	**AST**	**ALT**	**AKP**	**δGT**	**ALB**
	3.5-6.5 pmol/L	10.2-31.0 pmol/L	0.35-5.53 mU/L	3.4-17.1 Umol/L	<40 U/L	<58 U/L	40–130 U/L	<60 U/L	34–48 g/L
1^ST^ admission 2012-07-11	30.0	84.0	0.02	15.1	26.1	32.8	121.5	44.6	42.0
**2012-07-25 1st Dose of RAI**									
7-days post-RAI dose 1 2012-07-31				28.0	68.0	186.0	96.0	127.0	30.0
After 1-week of liver protection treatment 2012-08-06				19.4	25.9	54.0	95.4	107.5	32.0
**2012-09-02 2nd Dose of RAI**									
2nd Admission 2012-09-14	30.0	128.0	0.01	44.1	29.0	27.9	148.6	152.6	30.0
After 12-days of liver protection treatment 2012-09-25 Discharge	30.0	112.0	0.24	88.5	26.9	58.1	137.1	90.8	32.0

7-days after the first RAI dose of 5 mCi, the patient’s blood chemical tests revealed an abnormal liver function (Table 1), the abdominal ultrasound showed no abnormalities. The patient was treated with Essentiale Forte N® [5] (Polyene Phosphatidyl Choline) and Bicyclol® [6] for liver protection and prednisolone (30 mg qd).

One week later, the blood investigations showed marked recovery from the liver failure (Table 1).

About 5-weeks following the first RAI dose, the patient was subjected to his second dose of 5 mCi. 2-weeks following the second RAI dose, the patient presented to our department complaining of discomfort in the right upper quadrant, loss of appetite, palpitation and light coloured stools.

Upon admission, the blood tests revealed marked liver dysfunction (Table 1). Abdominal ultrasound showed no abnormalities. The patient was diagnosed with hepatotoxicity following the administration of a dose of RAI. The treatment regimen was aimed at liver protection with injections of Samyr® [7] (ademetionine-1, 4-butanedisulphonate) and Tianqing Ganmei® [8] (magnesium isoglycyrrhizinate); intravenous Vitamin C [9] administration, prednisolone (30 mg qd) and Leucogen® [10] (asparaginase) to raise the white cell count.

The liver toxicity was resolved within 10-days (Table 1). Upon discharge, the thyroid function test showed (Table 1). In an outpatient review, 2 months later, the patient thyroid function proved to be euthyroid and the liver function remained on the normal side.

### Case 2

A 34-years-old female patient was diagnosed 10-years ago with GD with history of tremors, heat intolerance, sweating, weight loss, increased appetite, irritability, increased stool frequency and reduced menstrual flow and was put on methimazole (5 mg qd). The patient self-discontinued the treatment and upon review, the thyroid hormones showed normalisation.

1-year after the discontinuation of medication, there was a relapse and the patient was admitted to her treating-hospital at the time. Upon discharge, the patient was put back on methimazole (2.5 mg qd). 10-months ago, the patient attended our out-patient department for review. The thyroid hormone levels showed a normalisation and the treating physician advised to stop all medications.

12-days following the discontinuation of treatment, the patient observed a recurrence of palpitation and tremors. The patient was referred to our department for a thyroid function test and the blood chemical tests documented normal renal function and hepatic values but revealed a significant increase in serum thyroid hormone levels (Table 2). A relapse of Grave’s hyperthyroidism was diagnosed and 2 days later, she was admitted as in-patient for treatment with RAI. The patient had good mental health, good sleeping pattern, good appetite, normal bowel frequency, normal urine and no significant recent weight change. She had no history of taking iodine-containing drug and no history of throat or neck pain. The possibility of primary liver disease could be ruled out; there was no evidence of immunological conditions (autoimmune hepatitis, primary sclerosing cholangitis or primary biliary cirrhosis); neither was metabolic liver disease due to alpha-1 antitrypsin deficiency, hemochromatosis or Wilson´s disease identified. Hepatitis serology was negative and serology for several other virus infections that might lead to liver dysfunction did not indicate presence of active virus infection. The patient´s alcohol consumption was sparse limited to less than one drink a month.

**Table 2:** Chronological thyroid function test and liver function test for case 2

**Case 2**									
**Event**	**fT3**	**fT4**	**TSH**	**Tb**	**AST**	**ALT**	**AKP**	**δGT**	**ALB**
	3.5-6.5 pmol/L	10.2-31.0 pmol/L	0.35-5.53mU/L	3.4-17.1 Umol/L	<40 U/L	<58 U/L	40–130 U/L	<60 U/L	34–48 g/L
1st admission 2011-12-05	16.67	53.15	0.02	4.8	37.0	52.0	58.0	26.0	75.0
**2011-12-08 Dose of RAI**									
2nd admission 2011-12-15	11.42	40.21	0.01	28.4	407.0	796.0	168.0	188.0	40.0
After 13-days of liver protection treatment 2011-12-28 Transfer to hepato-biliary department	9.38	44.1	0.09	130.8	59.0	29.0	111.0	54.0	38.0

Upon examination, she had 2nd degree bilateral thyroid enlargement, soft in consistency, non tender, no palpable nodules and no thyroid bruit on auscultation. Chest X-ray was normal, abdominal ultrasound showed no abnormalities, orbital CT showed bilateral exophthalmos; the prominence was of 19.1 mm and 18.8 mm Hertel readings on right and left sides respectively. Thyroid scan showed bilateral lobe swelling and radioactive Iodine uptake test showed a significantly elevated thyroid ^123^I uptake function at 2 and 24 hours of 40% and 59.0% respectively. She received RIT (10 mCi) and was discharged the following day on 30 mg of prednisolone per day.

5-days following the RAI treatment, the patient started complaining of a mild sense of nausea; she underwent serological investigations, which pointed towards a hepatic dysfunction (Table 2). The patient was readmitted, her liver function test showed hepatotoxicity (Table 2), abdominal ultrasound did not show any abnormal finding and the following treatment was administered: Atomolan® [11] (reduced glutathione tablets), Essentiale Forte N® [5] (polyene phosphatidyl choline injection) and Bifendate® [12] as liver protective treatment, prednisolone (30 mg qd) and Leucogen® [6] (asparaginase) to raise white blood cells.

13-days after the second admission, despite the intensive hepato-protective treatment, the patient’s liver function markers remained elevated with the exception of the alanine aminotransferase (ALT) (Table 2). The patient was eventually transferred to the Hepato-biliary department to continue her intensive treatment. 3-weeks later, her liver function tests were back to normal and she was ultimately discharged from the Hepato-biliary department. Till date, the liver function is normal and she is currently euthyroid.

## Discussion

The present patients had concomitant thyrotoxicosis and hepatic dysfunction from treatment with RAI. Both patients were investigated according to ordinary considerations concerning the cause of liver disease. In these cases, isolated liver dysfunction could be seen, which was in accordance with the liver enzymes that showed predominant signs of liver dysfunction. The possibility of primary liver disease could be ruled out as mentioned in the described cases above.

Liver injury caused by thyrotoxicosis is relatively common and can be conveniently divided into hepatitic or cholestatic types [13].

Iodine is an indispensable component of thyroid hormones levothyroxine (T4) and triiodothyronine (T3). Thyroid cells extract and concentrate iodide from plasma [14].

The liver is the primary organ of thyroid hormone metabolism. This partly explains the complexity of the influences of increased thyroid hormone levels on liver function tests. Individual differences are attributed to liver enzyme levels [15].

Hepatic radioiodine uptake was positively correlated with the dose of administered I-^131^ and increased levels of serum AST and ALT [16]. In our patients, several factors are able to potentiate the RAI-induced hepatotoxicity, i.e. age (adults are generally more susceptible to hepatotoxicity than children), sex (women are more commonly affected than men), nutritional factors (malnutrition). Moreover, we also noted a probable relationship of the period of disease (long standing Grave’s disease), dose-related (high dose of RAI), size of the goitre (both patients had very enlarged thyroid lobes) with the increased susceptibility to develop hepatotoxicity.

When hyperthyroidism due to GD persists after 6 months following 131I therapy, or if there is minimal response 3 months after therapy, retreatment with 131I is suggested [17].

## Conclusion

Although hepatic toxicity is a rather uncommon event after RAI treatment, physicians should be aware of this probable complication and consider starting an early hepato-protective treatment regimen prior to administration of RAI.

While there are controversies in treatment of thyrotoxicosis with RAI, with appropriate patient selection and regular follow-up, radioiodine is a safe and effective modality in achieving high cure rates [18].

## Consent

Written informed consent was obtained from the patients for publication of this case report. A copy of the written consent is available for review by the Editor-in-Chief of this journal.

## Abbreviations

GD: Graves’ disease; RAI 131I: Radioactive iodine; ATD: Anti-thyroid drug; NTR: Near-total resection; RIT: Radioiodine therapy; EHL: Effective half life; CT: Computer tomography; T4: Serum levothyroxine; f T4: Free serum levothyroxine; T3: Serum triiodothyronine; f T3: Free Serum triiodothyronine; TSH: Serum thyroid stimulating hormone; Tb: Serum total bilirubin; AST: Serum aspartate aminotransferase; ALT: Serum alanine aminotransferase; AKP: Serum alkaline phosphatase; δGT: Serum gamma glutamyl transpeptidase; ALB: Serum albumin

## Competing interests

The authors declare that they have no competing interests.

## Authors’ contributions

NPJ wrote the first draft. NPJ and BT were in a position of leadership for the patients and collected information on the patients. BT and NPJ did the literature searches. BT wrote the final manuscript and made appropriate revisions. QS supervised the writing of the manuscript. All authors read through and approved the final manuscript.
